# Supplementary material for: MetaRibo-Seq measures translation in microbiomes
Source: Nat Commun. 2020 Jun 29;11:3268. doi: 10.1038/s41467-020-17081-z (PMC7324362; doi:10.1038/s41467-020-17081-z)
Supplement: Supplementary file 10 — Supplementary Data 7 [file 41467_2020_17081_MOESM10_ESM.zip › File2/Confidence_VeryHigh_Taxonomy/11083_out.krona.html]

Javascript must be enabled to view this page.

members
magnitude
magnitudeUnassigned
count
unassigned
taxon
rank

11083\_out

4

superkingdom
2
4

1239
phylum
3

1
class
91061

1
order
186826

1
family
1300

genus
1301

SRS143872\_contig\_number\_contig-100\_6260.6260
1

186801
class
2

order
186802
2

species
1898207

SRS147271\_contig\_number\_contig-100\_5355.205967
1

1
186803
family

genus
265975
1

species
237576

SRS018875\_contig\_number\_23668
1

1
phylum
201174

1
class
1760

1
order
2037

1
2049
family

1
1654
genus

species
1660
1

SRS020856\_contig\_number\_contig-100\_2258.32926.32926
